# Optimal Mode of clearance in critically ill patients with Acute Kidney Injury (OMAKI) - a pilot randomized controlled trial of hemofiltration versus hemodialysis: a Canadian Critical Care Trials Group project

**DOI:** 10.1186/cc11835

**Published:** 2012-10-24

**Authors:** Ron Wald, Jan O Friedrich, Sean M Bagshaw, Karen EA Burns, Amit X Garg, Michelle A Hladunewich, Andrew A House, Stephen Lapinsky, David Klein, Neesh I Pannu, Karen Pope, Robert M Richardson, Kevin Thorpe, Neill KJ Adhikari

**Affiliations:** 1 Division of Nephrology, St. Michael's Hospital, 30 Bond Street, Toronto, ON, M5B 1W8, Canada; 2 Keenan Research Centre in the Li Ka Shing Knowledge Institute of St. Michael's Hospital, 30 Bond Street, Toronto, ON, M5B 1W8, Canada; 3 Critical Care and Medicine Departments, St. Michael's Hospital, 30 Bond Street, Toronto, ON, M5B 1W8, Canada; 4 Interdepartmental Division of Critical Care, University of Toronto, 30 Bond Street, Toronto, ON, M5B 1W8, Canada; 5 Division of Critical Care Medicine, University of Alberta, 8440 112 Street NW. Edmonton, AB, T6G 2B7, Canada; 6 Division of Nephrology, London Health Sciences Centre, 800 Commissioners Road East, London, ON, N6A 5W9, Canada; 7 Division of Nephrology, Sunnybrook Health Sciences Centre, 2075 Bayview Avenue, Toronto, ON, M4N 3M5, Canada; 8 Department of Critical Care Medicine and Sunnybrook Research Institute, Sunnybrook Health Sciences Centre, 2075 Bayview Avenue, Toronto, ON, M4N 3M5, Canada; 9 Division of Critical Care, Mt. Sinai Hospital, 600 University Avenue, Toronto, ON, M5G 1X5, Canada; 10 Applied Health Research Centre, Li Ka Shing Knowledge Institute, St. Michael's Hospital, 30 Bond Street, Toronto, ON, M5B 1W8, Canada; 11 Division of Nephrology, University of Alberta Hospital, 8440 112 Street NW, Edmonton, Alberta, T6G 2B7, Canada; 12 Division of Nephrology, University Health Network, 200 Elizabeth Street, Toronto, ON, M5G 2C4, Canada

## Abstract

**Introduction:**

Among critically ill patients with acute kidney injury (AKI) needing continuous renal replacement therapy (CRRT), the effect of convective (via continuous venovenous hemofiltration [CVVH]) versus diffusive (via continuous venovenous hemodialysis [CVVHD]) solute clearance on clinical outcomes is unclear. Our objective was to evaluate the feasibility of comparing these two modes in a randomized trial.

**Methods:**

This was a multicenter open-label parallel-group pilot randomized trial of CVVH versus CVVHD. Using concealed allocation, we randomized critically ill adults with AKI and hemodynamic instability to CVVH or CVVHD, with a prescribed small solute clearance of 35 mL/kg/hour in both arms. The primary outcome was trial feasibility, defined by randomization of >25% of eligible patients, delivery of >75% of the prescribed CRRT dose, and follow-up of >95% of patients to 60 days. A secondary analysis using a mixed-effects model examined the impact of therapy on illness severity, defined by sequential organ failure assessment (SOFA) score, over the first week.

**Results:**

We randomized 78 patients (mean age 61.5 years; 39% women; 23% with chronic kidney disease; 82% with sepsis). Baseline SOFA scores (mean 15.9, SD 3.2) were similar between groups. We recruited 55% of eligible patients, delivered >80% of the prescribed dose in each arm, and achieved 100% follow-up. SOFA tended to decline more over the first week in CVVH recipients (-0.8, 95% CI -2.1, +0.5) driven by a reduction in vasopressor requirements. Mortality (54% CVVH; 55% CVVHD) and dialysis dependence in survivors (24% CVVH; 19% CVVHD) at 60 days were similar.

**Conclusions:**

Our results suggest that a large trial comparing CVVH to CVVHD would be feasible. There is a trend toward improved vasopressor requirements among CVVH-treated patients over the first week of treatment.

**Trial Registration:**

ClinicalTrials.gov: NCT00675818

## Introduction

Acute kidney injury (AKI) is a common complication of critical illness, afflicting up to two-thirds of patients admitted to the ICU [1]. A significant minority of patients with AKI requires renal replacement therapy (RRT), and these individuals have high short-term mortality that ranges from 50 to 70% [2]. In an attempt to mitigate these poor outcomes, various components of the RRT prescription have been rigorously examined in large well-designed randomized controlled trials (RCTs) [3-5].

The optimal mode of clearance in patients with AKI who require renal support is an area of considerable controversy resulting in significant practice variation [6]. Hemofiltration, whereby solutes are removed by convection, facilitates the removal of both low and higher molecular weight solutes, depending on the pore size of the membrane [7]. Hemodialysis, in which solute removal occurs via diffusion out of the bloodstream into the dialysate down a concentration gradient, removes low molecular weight molecules but provides limited clearance of higher molecular weight substances. When filter characteristics are kept constant, hemofiltration, which more faithfully mimics glomerular filtration, should result in the clearance of larger-sized solutes as compared to hemodialysis [7]. The removal of such solutes, which may include toxic mediators of sepsis and inflammation, provides the theoretical underpinnings for the superiority of hemofiltration as a renal support mode for critically ill patients with AKI.

We conducted a multicenter pilot RCT of hemofiltration vs. hemodialysis in critically ill patients with AKI to determine whether a larger definitive trial based on clinically important endpoints would be feasible. In a secondary analysis, we evaluated whether hemofiltration improved global organ dysfunction.

## Materials and methods

### Study design

We conducted an unblinded RCT of continuous venovenous hemofiltration (CVVH) vs. continuous venovenous hemodialysis (CVVHD) with concealed allocation (http://Clinicaltrials.gov registration number NCT00675818). Our reporting follows the updated CONSORT statement [8].

### Setting

Participants were recruited from ICUs at six academic hospitals: Mt. Sinai Hospital (Medical-Surgical ICU), Sunnybrook Health Sciences Centre (Critical Care Unit and Cardiovascular ICU) and St. Michael's Hospital (Medical-Surgical and Cardiovascular ICUs), all in Toronto, Canada; Victoria Hospital (Critical Care Trauma Centre), and University Hospital (Medical/Surgical Intensive Care Unit and Cardiac Surgery Recovery Unit), both in London, Canada, and University of Alberta Hospital (General Systems Intensive Care Unit) in Edmonton, Canada. The Research Ethics Boards of Mt. Sinai Hospital, St. Michael's Hospital, Sunnybrook Health Sciences Centre, London Health Sciences Centre and the University of Alberta approved the protocol. The Applied Health Research Centre at St. Michael's Hospital (Toronto, Ontario, Canada) was the trial coordinating center.

### Population

We enrolled critically ill adults (≥ 16 years of age) with AKI, defined as a serum creatinine increase ≥ 50% from baseline (defined as the last known pre-morbid serum creatinine or earliest value available from the current admission). At the time of screening, at least one of the following indications for RRT initiation needed to be present: (i) oliguria (defined as urine output < 100 mL in the preceding 4 hours); (ii) metabolic acidosis (serum bicarbonate < 15 mmol/L and pH < 7.25); (iii) refractory hyperkalemia (serum potassium > 6 mmol/L despite medical efforts at potassium removal); (iv) serum urea > 50 mmol/L, or (v) suspected uremic organ involvement (pericarditis, encephalopathy, neuropathy or myopathy). Finally, participants needed to be hemodynamically unstable, defined as Sequential Organ Failure Assessment (SOFA)- Cardiovascular score ≥ 1 on the day of screening (see Additional file 1 for the modified SOFA score used in this study). This required the patient to have mean arterial pressure < 70 mmHg or receipt of at least one vasopressor or inotrope [9]. Patients were excluded if any one of the following was present: receipt of any RRT within the previous 2 months; presence of an obstructive etiology for AKI; receipt of a kidney transplant within the preceding year; diagnosis of rapidly progressive glomerulonephritis, vasculitis, or acute interstitial nephritis; a clinical indication for intermittent hemodialysis (for example, the presence of a dialyzable toxin); terminal illness with an associated life expectancy less than 2 months; moribund status (life expectancy < 48 hours as per judgment of physicians involved in the patient's care); prior enrolment in this study; enrolment in a competing ICU interventional study; non-availability of a CRRT machine, or administration of RRT for > 36 hours prior to eligibility assessment.

### Patient allocation

After eligibility was confirmed, we attempted to obtain consent from the patient or if the patient lacked capacity to consent, his/her substitute decision maker (SDM) was approached. A deferred consent option was approved at three sites, which allowed patient enrollment and randomization in the event of patient incapacity and the inability to locate an SDM. Using this mechanism, patients were randomized, and research personnel attempted to locate an SDM every 72 hours to affirm consent for participation. In all cases, when participants regained capacity, they were asked to provide consent if they were initially enrolled using *a priori *SDM consent or deferred consent; no participant withdrew consent once regaining capacity. Patients were allocated to a study group using sealed, opaque, sequentially numbered envelopes (prepared by the coordinating center) that were opened after consent was obtained [10]. Randomization was stratified by center in random blocks of four, six or eight. The Research Ethics Board at each center approved the study. An independent data and safety monitoring board tracked the trial's conduct.

### Study intervention

Participants randomized to CVVH were prescribed ultrafiltration with isovolemic replacement solution (evenly split between pre- and post-filter) to achieve a target clearance of 35 mL/kg body weight/hr. The prescribed hourly ultrafiltration rate was increased above 35 mL/kg/hr to compensate for the reduced efficiency of clearance related to the pre-filter component of the replacement solution volume administered. This adjusted CVVH dose was calculated from the post-filter replacement fluid (RF), pre-filter replacement fluid and blood (blood) flow rates as follows:

Dose = Postfilter RF rate + ((Prefilter RF rate × (Blood flow/(Blood flow + Prefilter RF rate))).

In the CVVHD arm, the dialysate flow was set to achieve a clearance of 35 mL/kg/hr, which included a post-filter hemofiltration flow of 100 to 200 mL/hr. This obligate low-volume post-filter hemofiltration is utilized at participating centers to minimize the risk of blood clotting in the machine's deaeration chamber. Ultrafiltration performed for achievement of net fluid balance or to compensate for the volume of administered anticoagulants (namely, citrate or unfractionated heparin) was not considered in the total dose. We used the patient's most recent measured body weight, as recorded in the chart or estimated by the study coordinator if no value was documented, to calculate prescribed RRT dose. Due to difficulties in achieving the target RRT dose as a result of high transmembrane pressures in some patients with higher body mass (particularly those randomized to CVVH), the protocol was modified in May 2010 (21 months after randomization of the first patient) such that the total fluid dose was capped at 4,000 mL/hr for both treatment arms, irrespective of the patient's weight. After this protocol change, twenty patients were enrolled in the trial; the 4,000 mL/hr dosing cap was invoked in six participants (five allocated to CVVHD and one to CVVH) who would have otherwise needed higher flows to achieve an actual total dose of 35 mL/kg/hr.

All study therapies were delivered by the Gambro Prismaflex™ RRT machine using the ST100 (surface area 1.0 m^2^) or ST150 (surface area 1.5 m^2^) filter sets, which contain a polyacrylonitrile AN69 membrane (Gambro, Richmond Hill, ON, Canada). We permitted the use of any commercially available dialysate and replacement solutions. Decisions regarding circuit anticoagulation (heparin, regional citrate anticoagulation, or no anticoagulation) and volume control were at the discretion of the attending physicians. Patients remained on study therapy until death, withdrawal of CRRT as part of withdrawal of life support, hemodynamic stability (SOFA-cardiovascular score < 2 for > 24 hrs) permitting stepdown to intermittent hemodialysis, or recovery of kidney function (defined as urine output > 500 mL in the preceding 12 hrs, and most recent serum potassium < 5.5 mmol/L and serum bicarbonate > 18 mmol/L).

### Outcomes

The primary feasibility outcome of this study was the ability to administer > 75% of the prescribed CRRT dose to participants in each treatment arm. Secondary feasibility outcomes included the ability to enroll > 25% of fully eligible patients and the ability to follow > 95% of patients to 60 days following randomization (the anticipated follow-up period for the future definitive principal study). Secondary outcomes included change in SOFA score from baseline to days 1, 2 and 7, respectively, following randomization. Serial changes in SOFA scores have been shown to be correlated with clinical outcomes in critically ill patients with AKI who require RRT [11].

### Data collection

Trained research coordinators collected baseline clinical and demographic data, and information on pre-existing medical conditions. Specific risk factors for AKI were ascertained, including recent procedures, nephrotoxins, and sepsis (defined using consensus guidelines [12]). SOFA score was calculated at the time of randomization and on each day of study therapy. The SOFA-Cardiovascular score was modified to include the receipt of vasopressin. Patients receiving RRT on a given day were assigned a SOFA-Renal score of 4, regardless of urine output or serum creatinine. Participants were followed until death or a maximum of 60 days from randomization, at which time vital status and the ongoing need for RRT among survivors were recorded.

### Statistical analyses

As this was a feasibility trial with the primary objective of informing the design of a large-scale RCT, we planned to enroll a convenience sample of 75 participants from six sites. Patients who were randomized but never received RRT are described, but these individuals were replaced to ensure that at least 75 patients received some form of RRT. Since the primary feasibility outcome was based on the dose of CRRT received, patients for whom dose could not be readily calculated (those who received no RRT or forms of RRT other than CRRT) were excluded from the analysis related to feasibility. However, clinical outcomes are reported for all randomized participants.

Descriptive statistics were used to characterize participants in either arm. Continuous variables are presented as means (SD) or medians (interquartile range, IQR) and two-group comparisons were performed with the *t*-test or Wilcoxon test, as appropriate. Two-group comparisons involving categorical variables were carried out with the chi square test. Analysis of covariance, adjusted for baseline SOFA score, was used to evaluate the change in SOFA score on days 1 and 2. Linear mixed models adjusted for baseline SOFA score and day of study therapy were used to evaluate the impact of RRT mode on SOFA score over the first week of therapy. For the fixed effect of treatment (that is, CVVH vs. CVVHD) 95% confidence intervals (CI) were obtained by profiling the log-likelihood function. All analyses were performed using R version 2.12.0 (R Development Core Team 2010, Vienna, Austria).

## Results

**Figure 1:**
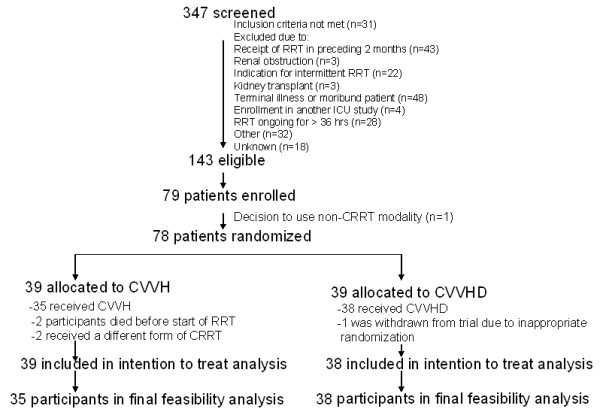
**Flow of patients through the trial**. RRT, renal replacement therapy; CRRT, continuous renal replacement therapy; CVVH, continuous venovenous hemofiltration; CVVHD, continuous venovenous hemofiltration

We screened 347 patients; 143 were eligible for participation and 79 individuals (55.2%) were enrolled over a 24-month time period. The inability to obtain consent from the patient or SDM was the reason for the non-enrollment of otherwise eligible patients. One patient was excluded shortly after enrollment after it was decided to pursue a non-continuous form of RRT. In total, 78 patients were randomized (39 to CVVH, and 39 to CVVHD). In one case, prior to the start of therapy it was recognized that a patient randomized to CVVHD was inappropriately enrolled as the indication for RRT was toxin removal rather than AKI *per se*. This patient was excluded from all further analyses. Clinical outcomes are reported in an intention-to-treat fashion for the remaining 77 patients (CVVH, 39; CVVHD, 38). Four patients randomized to CVVH were excluded from the feasibility analysis, two due to death prior to commencement of study RRT, and two due to receipt of continuous venovenous hemodiafiltration as the initial mode of therapy. The indication(s) for RRT was (were) oliguria, metabolic acidosis, hyperkalemia and uremia in 34, 17, 6 and 8 patients, respectively, in the CVVH arm. In the CVVHD arm, these indications guided the inclusion of 36, 15, 4 and 3 participants, respectively. In total, 73 participants commenced the therapy to which they were randomized (CVVH, 35; CVVHD, 38); these individuals contributed to the analysis relating to the feasibility of treatment delivery (Figure 1).

### Baseline characteristics (Table 1)

The mean age of participants was 61.5 (SD 14.2) years and 30/77 (39%) were women. The median time from ICU admission to randomization was 2 (IQR 2 to 8) days. Sepsis was present in 63/77 (82%) of participants, 73/77 (95%) were mechanically ventilated and 69/77 (90%) received vasopressors at the time of randomization. Median 24-hr urine output was 125 (IQR 50 to 250) mL. Approximately two-thirds of participants received some form of RRT prior to randomization, with 23/39 in the CVVH group (59%) for a mean of 10.6 (SD 12.0) hrs, and 30/38 in the CVVHD group (79%) for a mean of 13.3 (SD 11.7) hrs. (Table 1

**Table 1:** Baseline characteristics

	**CVVH (*n *= 39)**	**CVVHD (*n *= 38)**
Age, years	58.8 ± 13.7	64.3 ± 14.3
Female	14 (36%)	16 (42%)
Weight, kg	86.6 ± 31.5	89.2 ± 26.4
Days from ICU admission to randomization	2 (1, 4)	2 (1, 3)
SOFA score	16.3 ± 3.3	15.5 ± 3.0
**Premorbid conditions**		
Hypertension	19 (49%)	26 (68%)
Diabetes mellitus	9 (23%)	9 (24%)
Chronic kidney disease	5 (13%)	13 (34%)
**Acute kidney injury risk factors**		
Sepsis	31 (79%)	32 (84%)
Cardiopulmonary bypass in past 7 days	1 (3%)	4 (11%)
IV contrast in past 7 days	9 (23%)	6 (16%)
**Physiologic parameters**		
Urine output, mL/24 hr	125 (60, 245)	135 (46, 251)
Minimum systolic BP during 24 hr before enrolment, mmHg	94.2 ± 16.5	86.1 ± 13.5
Minimum diastolic BP during 24 hr before enrolment, mmHg	48.9 ± 8.6	47.2 ± 9.4
**Laboratory parameters**		
Serum creatinine, µmol/L	276 (194, 352)	246 (155, 325)
Urea, mmol/L	25.8 ± 38.1	17.6 ± 12.7
Potassium, mmol/L	4.3 ± 0.7	4.1 ± 0.6
Bicarbonate, mmol/L	20.5 ± 5.9	20.2 ± 5.3
Hemoglobin, g/L	86.1 ± 16.3	90.0 ± 15.2
Platelets, × 10^9^/L	109.6 ± 82.6	127.2 ± 80.9
WBC, × 10^9^/L	20.2 ± 43.3	16.9 ± 10.6
**ICU interventions**		
Duration of RRT prior to randomization, hours	8 (0, 19)	13 (1.0, 20.5)
Mechanical ventilation	37 (95%)	36 (95%)
Vasopressors	34 (87%)	35 (92%)
Total parenteral nutrition	1 (3%)	2 (5%)

Continuous data are displayed as mean ± SD or medians (interquartile range), as appropriate. Categorical variables are displayed as n (%). CRRT, continuous renal replacement therapy; CVVH, continuous venovenous hemofiltration; CVVHD, continuous venovenous hemodialysis; BP, blood pressure; RRT, renal replacement therapy; SOFA, Sequential Organ Failure Assessment; WBC, *White *blood cell.

### Features of study treatments (Table 2)

Among the 35 participants who started CVVH, RRT was prescribed for a median of 107 (IQR 55 to 146) hrs and delivered for 85 (IQR 43 to 128) hrs. Overall, 84.7% (95% CI 79.1, 90.3) of the prescribed duration was delivered. The mean weight-standardized dose was 33.6 (SD 7.4) mL/kg/hr while CRRT was ongoing.(Table 2

**Table 2:** Feasibility and safety data

	CVVH (*n *= 35)	CVVHD (*n *= 38)	*P*-value
Duration RRT prescribed, hrs	146 ± 240	145 ± 156	0.88
Duration RRT received, hrs	130 ± 222	128 ± 142	0.87
Mean pre-filter replacement solution flow, mL/hr	1533 ± 442	0	n/a
Mean post-filter replacement solution flow, mL/hr	1440 ± 488	180 ± 140	n/a
Mean dialysate flow, mL/hr	0	2871 ± 872	n/a
Mean RRT dose, mL/kg/hr	33.6 ± 7.4	34.7 ± 4.4	0.50
Prescribed dose delivered, %	84.7 ± 16.3	87.8 ± 13.7	0.73
Net ultrafiltration, L/day	1.7 ± 2.2	0.8 ± 4.1	0.98
Days on study therapy	5 (3-7)	4.50 (3.00-10.25)	0.79
**Primary reason for CRRT withdrawal**			0.75
Death while on CRRT	12 (35%)	10 (27%)	
Kidney function recovery	7 (21%)	7 (19%)	
Transfer to intermittent hemodialysis	12 (35%)	14 (38%)	
Withdrawal of life support	3 (9%)	6 (16%)	
Catheter changes/day of therapy	0.09 ± 0.2	0.1 ± 0.2	0.92
Units RBCs transfused/day of therapy	0.3 ± 0.3	0.4 ± 0.7	0.38
Unscheduled circuit changes/day of therapy	0.2 ± 0.3	0.2 ± 0.2	0.36
Study days with receipt of norepinephrine, %	61.8 ± 36.2	65.7 ± 7.5	0.41
Study days with receipt of vasopressin, %	35.4 ± 35.9	43.0 ± 43.6	0.51

Continuous data are displayed as mean ± standard deviation or median (interquartile range), as appropriate. Categorical variables are displayed as n (%); n/a, not applicable. Data are restricted to patients who initiated therapy with the CRRT mode to which they had been allocated. RRT, renal replacement therapy; CRRT, continuous renal replacement therapy; RBCs, red blood cells; CVVH, continuous venovenous hemofiltration; CVVHD, continuous venovenous hemofiltration.

CVVHD was prescribed in 38 participants for a median of 92 (IQR 57 to 145) hrs and delivered for 76 (IQR 44 to 148) hrs. Over the course of the trial, 87.8% (95% CI 83.3, 92.3) of the prescribed duration was delivered to patients randomized to receive CVVHD. The mean weight-standardized delivered RRT dose was 34.7 mL/kg/hr (SD 4.4).

### Clinical outcomes

All subjects were followed to 60 days, by which point 22/39 (56%) and 21/38 (55%) of participants assigned to CVVH and CVVHD, respectively, had died. Among surviving patients, 4/17 (24%) and 3/17 (19%) of those initially assigned to CVVH and CVVHD respectively, were still dependent on RRT.

**Figure 2:**
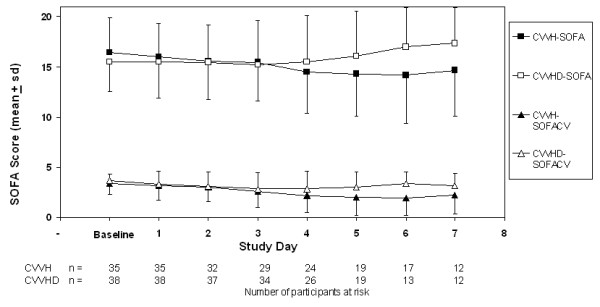
**Total and Cardiovascular (CV) Sequential Organ Failure Assessment (SOFA) scores over the first seven days following randomization**. Total SOFA scores are denoted with squares; the cardiovascular component of the SOFA score is denoted with triangles.CVVH, continuous venovenous hemofiltration; CVVHD, continuous venovenous hemofiltration

After adjustment for baseline SOFA score, we found a non-significant decline in SOFA score among participants treated with CVVH compared to CVVHD on the first day (-0.4, 95% CI -1.3, 0.6) and second day (-0.4, 95% CI -1.6, 0.8) following randomization. Over the first week of therapy, the adjusted change in the SOFA score among participants treated with CVVH compared to CVVHD was -0.8 (95% CI -2.1, 0.5). The observed reduction appeared to be driven by a reduction in the cardiovascular component of the SOFA score (Figure 2).

### Protocol violations and adverse events

We identified 15 protocol violations involving 15 participants. In one individual who started therapy with CVVH, achievement of the target dose was not feasible and RRT was supplemented with the addition of continuous dialysis; ongoing difficulties with maintaining adequate small molecule clearance using CRRT resulted in a change to sustained low efficiency dialysis. In five cases (one patient on CVVH, and four on CVVHD), stepdown from CRRT to IHD occurred before the participant met the study-defined criteria for hemodynamic stability. In five cases (two patients on CVVH, and three on CVVHD), RRT was discontinued altogether in participants who did not meet study criteria for renal recovery.

No adverse events were attributed to the study intervention in either treatment arm.

## Discussion

We completed a multi-center, concealed-allocation, randomized trial comparing hemofiltration and hemodialysis in critically ill patients with AKI. This pilot trial achieved its primary objective of confirming the feasibility of performing a large-scale trial evaluating RRT clearance mode in AKI. There was also a non-significant trend towards reduced organ dysfunction, driven by decreased vasopressor requirements, early after the initiation of RRT in patients who received hemofiltration. These feasibility data and the importance of the clinical question justify the conduct of a large trial with adequate power to evaluate the primary outcome of 60-day mortality. With a power of 0.80, type I error of 0.05, and a mortality reduction of 10% in patients treated with CVVH (estimated 60-day mortality in the CVVHD arm 55%), we estimate that such a trial would require the enrolment of nearly 400 patients per arm. A more conservative mortality reduction of 7.5% would require the enrolment of 700 patients per arm.

Little is known about the optimal mode of clearance in renal replacement for AKI. Although both convection and diffusion remove small molecules with equal efficiency, convection may remove larger molecules that are not cleared by diffusive mechanisms [13]; previous studies have examined effects on inflammatory markers and have been too small to reliably determine effects on clinical outcomes. In addition, the profile of larger molecules removed by convection is relatively non-specific and may also include molecules that dampen inflammation or crucial medications such as antibiotics [14]. A randomized crossover study of 13 patients with AKI and the systemic inflammatory response syndrome found that CVVH for 24 hrs reduced plasma concentrations of TNFα and cleared more IL-6, compared to CVVHD. However, there was no effect on plasma concentrations of IL-6, IL-10, SL-selectin, or endotoxin [15]. Morgera and coworkers randomized 24 patients with sepsis-associated AKI to treatment with CVVH or CVVHD using a high cutoff membrane permeable to molecules up to 60 kilodaltons in size. Plasma concentration and clearance for IL-6 did not differ, but clearance of IL-1 receptor antagonist, an anti-inflammatory mediator, was enhanced by CVVH [16]. Of note, protein losses were higher in patients who received CVVH. In a prospective crossover study involving 15 patients with AKI who sequentially received CVVH and CVVHD, β_2_-microglobulin clearance was non-significantly higher among CVVH recipients (*P *= 0.055) [17]. Among trials focusing on clinical outcomes, a single-centre RCT (n = 20) did not demonstrate a difference in survival, renal recovery or ICU stay in patients treated with CVVH vs. CVVHD administered at fixed doses of 1.7 to 2.0 L/hr [18]. Similarly, a recent unpublished 65-patient RCT of CVVH vs continuous venovenous hemodiafiltration (both at 40 mL/kg/hr) did not demonstrate a survival difference at 28 days [19].

While interventional trials involving devices and processes of care that are susceptible to large variations in practice are challenging, we achieved our feasibility objectives. Specifically, we were able to recruit the majority of eligible subjects, implement the protocolized therapy for > 85% of the prescribed time and ascertain vital status at 60 days for all participants. When accounting for actual time on therapy, the delivered dose exceeded 80% of that prescribed in both treatment arms, thereby surpassing our feasibility threshold of 75%. Accordingly, we believe our study strongly supports the feasibility of a large definitive randomized trial comparing hemofiltration and hemodialysis in critically ill patients with severe AKI.

This is the largest published trial to date to study the mode of solute clearance in AKI. Given the challenges of recruiting participants and implementing interventions in a population with a high burden of illness, the success of our pilot was a necessary precursor to a principal trial that examines patient-relevant clinical outcomes. Our eligibility criteria were pragmatic and assured the inclusion of individuals in North America who typically receive CRRT. A minority of individuals for whom no SDM could be found were enroled with deferred consent, thereby limiting exclusion of potentially eligible patients and mitigating selection bias. Of interest, no subject enroled by deferred consent had an SDM who subsequently withdrew consent or withdrew consent personally after regaining capacity. Finally, other than the clearance mode, all other aspects of RRT including stepdown to intermittent hemodialysis and withdrawal of RRT were performed in a manner consistent with usual practice.

Our study has several limitations. As this was an unblinded trial, we cannot exclude the effect of co-interventions in either treatment arm. However, the nature of our intervention made blinding impractical, and we ensured that the two groups received equivalent RRT doses. There is also no definite intervention related to RRT prescription that has been shown to modify outcomes in critically ill patients with AKI. In addition, we cannot exclude the possibility that patients who were eligible but not randomized were systematically different than trial participants. Our protocol specified a target clearance of 35 mL/kg/hr, which was generally achieved in both arms. The decision to use this dose was guided by the fact that our trial was designed when higher dose CRRT was felt to be potentially beneficial based on data from two trials [20,21]. This pre-dated more recent trials and meta-analyses demonstrating no advantage of higher dose CRRT over doses of 20 to 25 mL/kg/hr, which would likely be the standard in a future trial comparing convection and diffusion [4,5,22,23]. However, since high dose therapy appears safe and may positively modify the effect of convective clearance [20], our higher dose target was reasonable. It should be noted that while 35 ml/kg/hr was prescribed to all participants, actual solute clearance may have been significantly lower due to changes in filter permeability [24,25]. On the other hand, when calculating the prescribed dose, we did not consider clearance associated with net ultrafiltration or removal of volume associated with the administration of anticoagulants. This approach would tend to underestimate the actual solute clearance that was delivered. Importantly, any deviation in dose from our 35 mL/kg/hr target would affect participants in both intervention arms to a similar extent. The Prismaflex CRRT system requires that all patients (even those predominantly getting hemodialysis) receive a small amount of post-filter hemofiltration (up to 200 ml/hr) to prevent clotting in the machine's deaeration chamber. In addition, CVVHD recipients would have had other unavoidable sources of convective clearance, specifically for the achievement of net fluid removal, and for the isovolemic removal of volume associated with the administration of the anticoagulant (citrate or heparin). Thus, CVVHD recipients did not receive purely diffusive solute clearance. We nonetheless estimate that the typical patient enrolled in the CVVHD arm still received > 80% of therapy in the form of diffusive clearance, which reassures us that this trial truly compared two different modes of solute clearance. Our trial was conducted using the AN69 polyacryonitrile filter, which has unique adsorptive characteristics [14]. While this is a widely used membrane in the administration of CRRT, the adsorptive characteristics of filters may differ and we cannot generalize our findings to settings in which other filters are utilized. Moreover, we did not collect data to evaluate the relative effect of CVVH or CVVHD on the removal of molecules of varying size. This trial was conducted using continuous RRT. Although the question of hemodialysis vs. hemofiltration is applicable to patients stable enough to receive intermittent RRT, machines to deliver intermittent hemofiltration were not widely available in North America when the trial was conducted. Among trial participants who became more hemodynamically stable, intermittent hemodialysis was utilized even when the initial CRRT mode was hemofiltration, thereby potentially diluting the benefits associated with CVVH. Finally, while we observed a trend towards improved organ failure scores, this finding must be interpreted with caution given the small sample size of our trial. Moreover, disease severity scores such as the SOFA score are surrogate markers that cannot supplant hard clinical endpoints.

## Conclusions

Our findings clearly support the feasibility of performing a definitive trial comparing CVVH and CVVHD in critically ill patients with AKI. The early non-significant trend towards reduced vasopressor requirements provides preliminary support to the concept that convective modes of clearance reduce inflammation and thus benefit critically ill patients. Given the high mortality associated with AKI, the lack of specific RRT interventions shown to reduce mortality, and current practice variation, the results of our pilot trial provide justification for a larger trial of hemofiltration vs. hemodialysis that will be adequately powered to evaluate meaningful clinical outcomes.

## Key messages

• A randomized controlled trial of hemofiltration vs hemodialysis is feasible.

• Hemofiltration may be associated with decreased vasopressor requirements over the first week of therapy.

• A well-designed and adequately powered trial of hemofiltration vs hemodialysis would address an important area of uncertainty in the management of patients with AKI.

## Abbreviations

AKI: acute kidney injury; CRRT: continuous renal replacement therapy; CVVH: continuous venovenous hemofiltration; CVVHD: continuous venovenous hemodialysis; IL: interleukin; IQR: interquartile range; RF: replacement fluid; RCT: randomized controlled trial; RRT: renal replacement therapy; SDM: substitute decision maker; SOFA: Sequential Organ Failure Assessment; TNF α: tumor necrosis factor alpha.

## Competing interests

Dr. Bagshaw has served on a Gambro Experts Panel. No other relevant competing interests are reported.

## Authors' contributions

RW conceived and designed the study, obtained funding, supervised study conduct, interpreted the data, drafted the manuscript, and provided critical review of the manuscript. JOF conceived and designed the study, supervised study conduct, interpreted the data, drafted the initial version of the manuscript, and provided critical review of the manuscript. SMB interpreted the study data, supervised study conduct, and provided critical review of the manuscript. KEB interpreted the study data, supervised study conduct, and provided critical review of the manuscript. AXG interpreted the study data, supervised study conduct, and provided critical review of the manuscript. MAH interpreted the study data, supervised study conduct, and provided critical review of the manuscript. AAH interpreted the study data, supervised study conduct, and provided critical review of the manuscript. SL interpreted the study data, supervised study conduct and provided critical review of the manuscript. DK interpreted the study data, supervised study conduct, and provided critical review of the manuscript. RMR interpreted the study data, supervised study conduct, and provided critical review of the manuscript. NIP interpreted the study data and provided critical review of the manuscript. KP participated in data analysis and interpretation, designed data collection tools, and participated in the critical review of the manuscript. KT participated in study design, conducted the data analysis, participated in data interpretation, and in the critical review of the manuscript. NKJA conceived and designed the study, supervised study conduct, interpreted the data, drafted the initial version of the manuscript, and provided critical review of the manuscript. All authors approved the final version.

## Supplementary Material

Additional file 1 **Modified Sequential Organ Failure Assessment (SOFA) score**. SOFA score modified from the original version (see reference [9]) for application in the OMAKI trial. Click here for file
