# Supplementary material for: Optimal Mode of clearance in critically ill patients with Acute Kidney Injury (OMAKI) - a pilot randomized controlled trial of hemofiltration versus hemodialysis: a Canadian Critical Care Trials Group project
Source: Crit Care. 2012 Oct 24;16(5):R205. doi: 10.1186/cc11835 (PMC3682309; doi:10.1186/cc11835)
Supplement: Additional file 1 — Modified Sequential Organ Failure Assessment (SOFA) score. SOFA score modified from the original version (see reference [9]) for application in the OMAKI trial. [file cc11835-S1.DOC]

**Additional file 1. Modified Sequential Organ Failure Assessment (SOFA)**

**used in the OMAKI Trial**

**Score assigned based on the most extreme result for each category during the period of assessment.**

| | SOFA Score | 0 | 1 | 2 | 3 | 4 | | --- | --- | --- | --- | --- | --- | | ***Respiration***  **PaO_2_/FiO_2_** | ≥ 400 | ≤ 400 ≤ 300  (± resp. support) | | ≤ 200 ≤ 100  (+ resp. support) | | | ***Coagulation***  **Platelets(x 10^9^/L)** | ≥150 | ≤ 150 | ≤ 100 | ≤ 50 | ≤ 20 | | ***Liver***  **Bilirubin (µmol/L)** | < 20 | 20-32 | 33-101 | 102-204 | > 204 | | **Cardiovascular** | MAP ≥  70 mmHg | MAP < 70 mmHg | DA ≤ 5 µg/kg/min or dobutamine (any dose) | DA > 5 µg/kg/min or  EPI ≤ 0.1 µg/kg/min or NE ≤0.1  µg/kg/min  or VP ≤ 0.03 U/min | DA > 15 µg/kg/min or EPI > 0.1 µg/kg/min or NE > 0.1 µg/kg/min or VP >  0.03 U/min | | ***CNS***  **Glasgow Coma Scale** | 15 | 13-14 | 10-12 | 6-9 | < 6 | | ***Renal****  **Creatinine (µmol /L)** | < 110 | 110-170 | 171-299 | 300-440 or urine output **≥** 200 mL/d | ≥ 440 or urine output < 200 mL/d | |
| --- | --- | --- | --- | --- | --- | --- | --- | --- | --- | --- | --- | --- | --- | --- | --- | --- | --- | --- | --- | --- | --- | --- | --- | --- | --- | --- | --- | --- | --- | --- | --- | --- | --- | --- | --- | --- | --- | --- | --- | --- | --- | --- |

**DA= dopamine EPI= epinephrine NE= norepinephrine VP = vasopressin**

*** If patient received any form of renal replacement therapy during the day of assessment, an automatic Renal score of 3 or 4 is assigned. Serum creatinine is not considered when assigning the SOFA-Renal score if renal replacement was given on that day. If urine output is < 200 mL/ day, a score of 4 is given. If urine output is ≥ 200 mL/day, a score of 3 is given.**

**SOFA SCORE (total 0-24):** ______________
